# An unusual case of tuberculous spondylodiscitis in a patient with multiple neck masses

**DOI:** 10.1259/bjrcr.20210079

**Published:** 2022-02-21

**Authors:** Ammaarah Said, Ramya Balachandar, Niels van Vucht

**Affiliations:** 1 Department of Radiology, University College London Hospitals NHS Foundation Trust, London, United Kingdom

## Abstract

Tuberculosis is commonly thought of as a disease of the past or a disease of the developing world and immunocompromised populations. Resurgence in non-endemic populations has been trending in recent years. Although musculoskeletal manifestation of tuberculosis is less common—it has insidious onset and it is an indolent process, which in advanced stages can present with extensive pathology and severe morbidity. Diagnosis is often made by a combination of clinical features and imaging findings to initiate early treatment and to reduce complications such as vertebral collapse and cord compression. It is therefore vital for radiologists to be aware of imaging features and unusual presentations related to this destructive disease. This case report illustrates an unusual presentation in a young immunocompetent patient who presented with palpable neck masses and was later found to have extensive multilevel tuberculous spondylodiscitis. The salient features of this uncommon but debilitating disease are discussed and learning points highlighted.

## Clinical presentation

A 33-year-old male patient was referred by his GP to the Head and Neck clinic after noticing multiple areas of left neck swelling over 3 months. The patient reported an associated 1-year history of neck stiffness and shooting pain in his right arm; denying any back pain, lower limb, bowel or bladder symptoms or any generalised constitutional symptoms. The patient was unaware of his immunisation status, denied any past medical history and reported having lived in Iraq before coming into the UK.

On examination, bilateral non-tender enlarged anterior cervical lymph nodes and pain on passive cervical flexion and extension were elicited but no palpable bony, spinal or paraspinal tenderness. Clinical examination was otherwise normal with no neurological deficit.

The Head and Neck team proceeded with a routine neck ultrasound which demonstrated a large cystic retropharyngeal mass and associated enlarged lymphadenopathy. Ultrasound-guided fine needle aspiration of the cystic mass was performed and an urgent MRI whole spine requested.

Image-guided diagnosis of extrapulmonary TB was made and the patient commenced on oral antituberculous therapy. Ultrasound-guided neck lymph node aspiration later revealed mycobacterium tuberculosis complex on polymerase chain reaction. Sputum and blood cultures remained negative on acid fast bacteria culture (AFB) and polymerase chain reaction (PCR).

**Figure 1.**
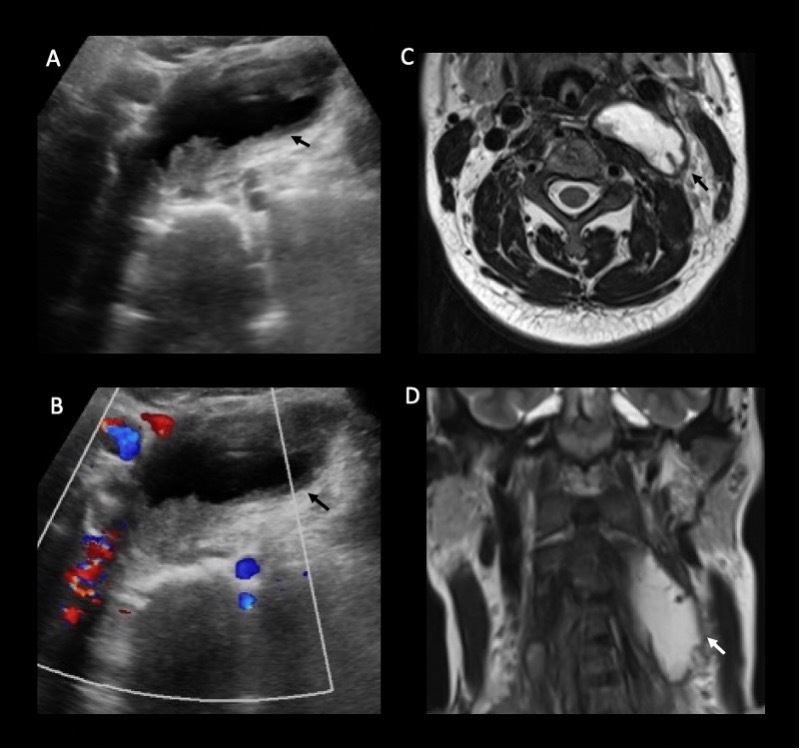
Images A and B are transversely oriented ultrasound images demonstrating a large left predominantly cystic retropharyngeal abscess measuring 8 × 4 x 2 cm (arrows). Images C and D, both *T_2_* weighted MRI sequences, demonstrate this retropharyngeal abscess within its left paravertebral location.

**Figure 2.**
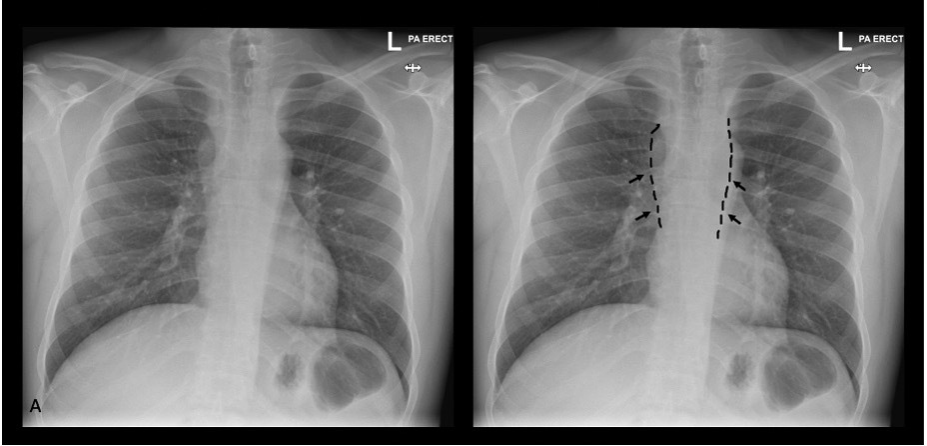
Image A and annotated image B are posteroanterior erect chest radiographs showing soft tissue density bulging along the right and left paravertebral lines (delineated on image B, note dotted lines and black arrows) representing underlying paraspinal abscesses seen on MRI.

**Figure 3.**
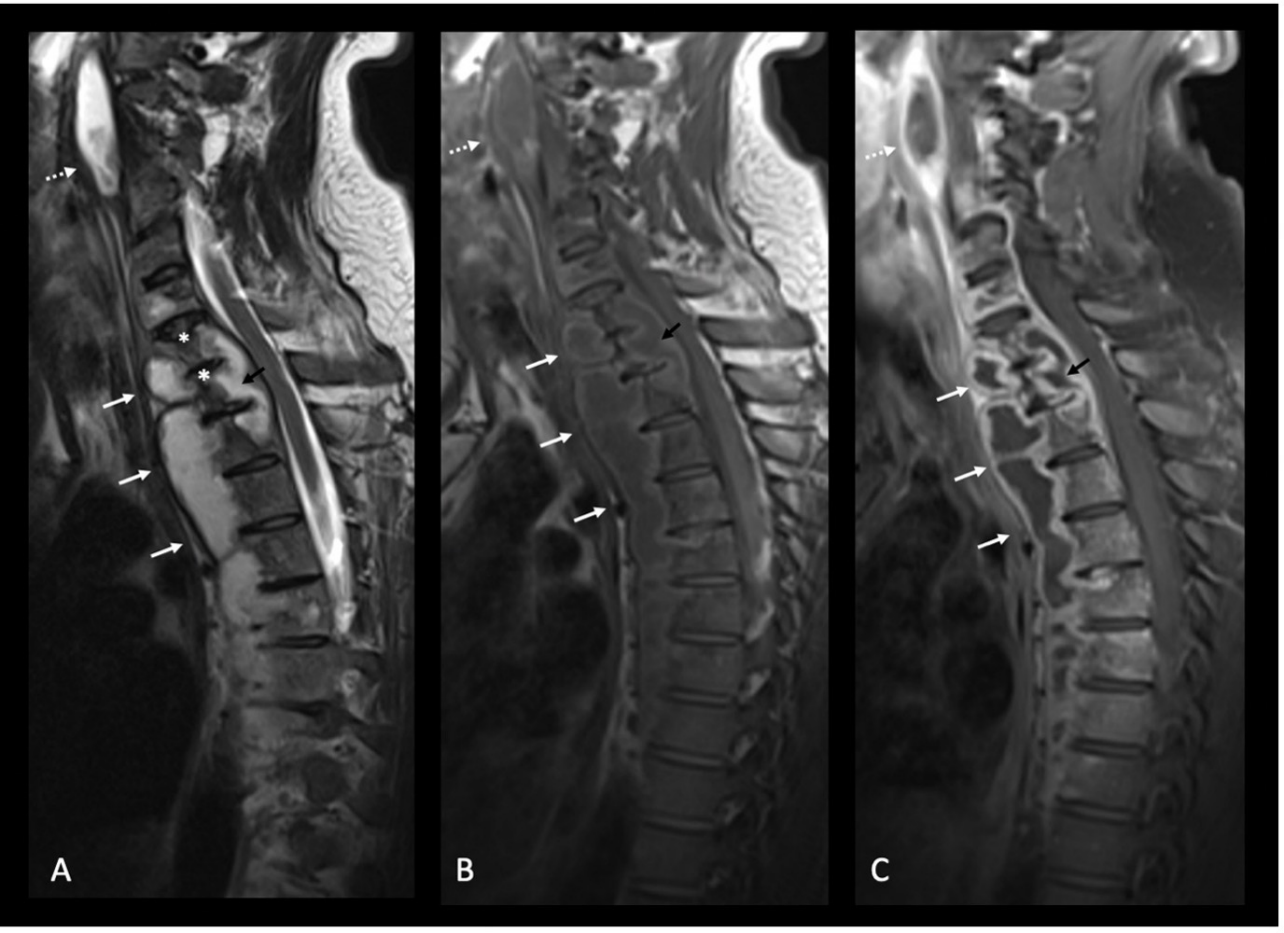
Sagittal *T_2_* weighted sequence (Image A), sagittal *T_1_* weighted sequence (Image B) and sagittal post-contrast T1 fat saturated sequence (Image C). Anterior paraspinal abscesses are demonstrated along the C7 to T9 vertebral levels (white arrows) and posterior paraspinal abscess along the T1 to T2 vertebral levels (black arrows), demonstrating T2 hyperintensity, T1 low signal and smooth peripheral contrast enhancement with bulging of the anterior and posterior longitudinal ligaments. Note that the intervertebral discs are largely spared, which is characteristic for tuberculous spondylitis in contrast to pyogenic spondylitis. Bony destruction with loss of anterior vertebral height at the C7 and T1 vertebra on Image A (asterisks) is subtly demonstrated and likely precedes impending Gibbus deformity if left untreated. Narrowing of the spinal canal at the T1/T2 vertebral levels (black arrow) is present. The left paravertebral retropharyngeal abscess is also noted anterior to the cervical spine at C2–C4 levels (dotted white arrows) and follows imaging characteristics in line with the paraspinal abscesses at the below.

**Figure 4.**
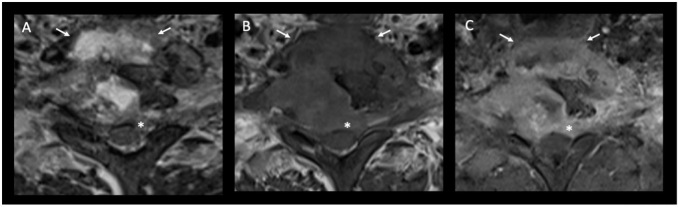
Transverse cross-sectional images at the T1 vertebral level, Image A is a *T_2_* weighted sequence, Image B is *T_1_* weighted and Image C post-contrast T1 fat saturated. White arrows point to the large paraspinal abscess which demonstrates high signal on T2, central low T1 signal on pre-contrast T1 and peripheral enhancement on post-contrast T1. This abscess extends from under the anterior longitudinal ligament to under the posterior longitudinal ligament, extending to form an extradural component which exerts mass-effect, narrowing the spinal canal and mildly compressing the spinal cord (asterisk).

**Figure 5.**
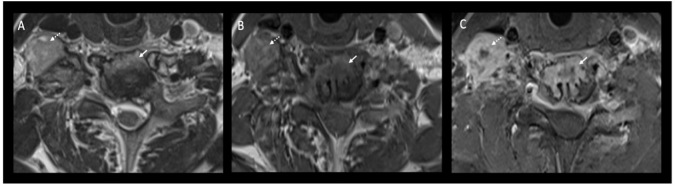
Transverse cross-sectional axial images of the cervical spine at the C6–C7 vertebral level. *T*_2_ weighted sequence (Image A), pre-contrast *T*_1_ weighted sequence (Image B) and post-contrast *T*_1_ weighted fat saturated sequence (Image C). A heterogenous, enhancing enlarged lymph node with central caseation is seen in the right anterior cervical chain (dotted white arrows) and an enhancing paraspinal soft tissue mass infiltrating the adjacent vertebral body with bony destruction is demonstrated (white arrow).

**Figure 6.**
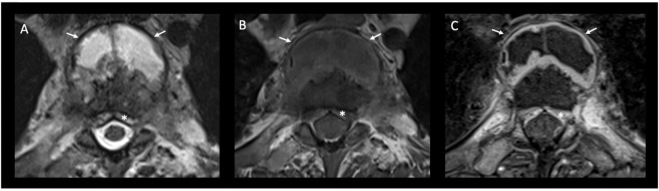
Transverse cross-sectional axial images at the cervicothoracic junction. *T*_2_ weighted sequence (Image A), pre-contrast *T*_1_ weighted sequence (Image B) and post-contrast T1 fat saturated sequence (Image C). The anterior paraspinal mass with high T2 signal, low T1 signal and peripheral contrast enhancement is again demonstrated (white arrows) as is the extradural soft-tissue extension (asterisk).

Below are series of radiological investigations performed on clinical presentation (Figures 1–6) including ultrasound, MRI head and neck and chest radiograph, illustrating classical imaging features of tuberculous spondylodiscitis.

## Imaging

### Differential diagnoses

Characteristic imaging features as demonstrated in this case allow for early image-based diagnosis, however, other spinal pathology can mimic TB spondylodiscitis.

Pyogenic spondylodiscitis is an important differential, although has a predilection for the lumbar spine and propensity to affect a spinal segment. Less commonly in immunosuppressed patients, Brucella, Coccidiomycosis and Aspergillosis may present similarly with multilevel involvement and subligamentous abscesses. Malignancy both primary and secondary can also present with bony destruction, abnormal bone signal and paraspinal soft tissue masses although contiguous multilevel involvement, abscess formation and disc space involvement are uncommon.

### Treatment and outcome

After an initial short in-patient stay, the patient was discharged to community care after starting on oral antituberculous therapy. Patient was followed up in his local hospital on a regular basis. He showed clinical and radiological improvements. Clinically, patient reported resolution of palpable neck lumps after several months post-treatment and did not report any pre-existing or new neurology.

Follow-up MRI performed 8 months post-treatment initiation demonstrated resolution of the epidural and prevertebral abscesses, reduction in the extent of vertebral levels involved (spanning C6-T5 compared to C2-T9 at presentation) with residual disease burden predominantly confined to the vertebral bodies.

Although the patient had subtle impending gibbus deformity on initial imaging, early diagnosis and treatment prevented further bony destruction and lifelong spinal deformity.

## Discussion

TB caused by *Mycobacterium tuberculosis* is a leading cause of worldwide morbidity and mortality. Globally, TB represents the 13th leading cause of mortality and was the second most common cause of death from a single infectious agent after Covid-19 in 2020.^1^ Musculoskeletal disease accounts for 1–3% of all extrapulmonary TB, of which TB spondylodiscitis also known as Potts disease makes up approximately 50%.^2^

Radiological diagnosis such as in this case is crucial and several imaging patterns of spinal TB have been described. The thoracolumbar junction and thoracic spine are most frequently involved, followed by the lumbar and then the cervical spine.^3^ Sacroiliac joint involvement has been reported in up to 9.7% of patients with spinal TB.^4,5^

Infection tends to begin in the anterior aspect of the vertebral bodies at the superior or inferior corners^6^ with classical subligamentous abscess formation—a hallmark of Spinal TB. These are known as ‘cold abscesses’ due to their tendency to spread extensively without much inflammatory change, distending the anterior and posterior longitudinal ligaments without penetration and tending to span multiple vertebral levels (usually more than three vertebrae^7^). Extension into the head and neck spaces or retroperitoneal cavity may be present with minimal clinical symptoms, as in this case where unusual neck lumps unveiled a more extensive destructive spondylodiscitis.

TB abscesses tend to demonstrate increased T2, decreased T1 signal and peripheral enhancement if contrast is administered. Abscess calcification and smooth thin walls are also specific, as is sparing of the intervertebral discs in early disease. Comparatively, pyogenic spondylitis involves the intervertebral discs earlier, with loss of disc height and herniation as well as forming abscesses with thick, irregular walls.

Loss of vertebral body cortical definition or scalloping of the anterior or posterior vertebral bodies known as the ‘gouge defect’ may be present. Vertebral body destruction and lack of sclerosis or subperiosteal reaction may be seen. This can lead to the classic ‘gibbus deformity’ with destruction of the anterior aspects of the vertebral body forming a kyphotic deformity and severity correlating with the number of vertebral bodies involved.^4^ Complete destruction of the vertebral bodies and concentric collapse may lead to vertebral plana. Increased vertebral body density forming an ‘ivory vertebra’ may be present indicating necrotic and non-viable bone.

Atypical multilevel non-contiguous skip lesions involving two non-contiguous vertebrae without any destruction of adjacent vertebral bodies or discs may be seen^8^ and highlights the importance of imaging the entire spine and sacroiliac joints.

Undetected and untreated, TB spondylodiscitis can lead to many complications. Thoracic spread of disease can cause destruction of the costovertebral joints and posterior ribs. Cranial spread can lead to retropharyngeal or neck space abscesses as demonstrated in this case and may lead to symptoms of dysphonia, dysphagia or difficulty breathing. Caudal spread can lead to psoas abscesses and descent below the inguinal ligament can present as abscesses in the groin, thigh, or gluteal region. Extradural extension also seen in this case can lead to cord compression ensuing in myelopathy or radiculopathy and rarely progression to tuberculous meningitis, myelitis, or intracranial extension with pachymeningeal disease and tuberculomas may occur.^8^

## Learning points

1. Tuberculous spondylodiscitis is a slow growing, indolent disease which usually manifests in advanced stages with irreparable damage to the spine and central nervous system. Awareness of the disease and its characteristic imaging features are important to the radiologist for early diagnosis and prevention of severe morbidity.
2. MRI of the spine is the preferred imaging modality to diagnose and assess extent and complications of tuberculous spondylodiscitis including cord compromise.
3. Whole spine MRI including the sacroiliac joints should be performed to assess for atypical multifocal skip lesions and tuberculous involvement of the sacroiliac joints.
4. Antituberculous therapy can be started based on imaging findings alone, prior to biochemical confirmation—as was the case with our patient and highlights the importance of appropriate imaging and awareness of spinal TB imaging features.
